# ATENA–A Novel Rapidly Manufactured Medical Invasive Ventilator Designed as a Response to the COVID-19 Pandemic: Testing Protocol, Safety, and Performance Validation

**DOI:** 10.3389/fmed.2021.614580

**Published:** 2021-08-19

**Authors:** Tiago Rebelo, Elizabete Neutel, Eurico Castro Alves, Francisco Barros, Hélder Oliveira, Humberto Machado, Joana Mendonça, João Fortuna Araújo, João Luís, José M. Pêgo, José Silva, Manuel Oliveira, Nuno Sousa, Paulo Figueiredo, Pedro Barata, Raquel Silva Magalhães, Rui Miguel Magalhães, Sara H. Gomes

**Affiliations:** ^1^CEiiA-Centre of Engineering and Product Development, Matosinhos, Portugal; ^2^Serviço de Anestesiologia, Centro Hospitalar Universitário Do Porto, Porto, Portugal; ^3^Departamento de Cirurgia, Centro Hospitalar Universitário Do Porto, Porto, Portugal; ^4^Center for Innovation, Technology and Policy Research (IN+), Instituto Superior Técnico, University of Lisbon, Lisbon, Portugal; ^5^Life and Health Sciences Research Institute, School of Medicine, University of Minho, Braga, Portugal; ^6^Instituto Ciências da Vida e da Saúde in Portuguese (ICVS)/3B's-PT Government Associate Laboratory, Braga, Portugal; ^7^Faculty of Health Sciences, University Fernando Pessoa, Porto, Portugal; ^8^Institute for Research and Innovation in Health, University of Porto, Porto, Portugal; ^9^Centro Hospitalar de Trás-os-Montes e Alto Douro, Vila Real, Portugal; ^10^Clinical Academic Center, Hospital of Braga, Braga, Portugal

**Keywords:** COVID-19, ATENA, pandemic, ventilator, safety, performance

## Abstract

**Background:** The urgent need for mechanical ventilators to support respiratory insufficiency due to SARS-CoV-2 led to a worldwide effort to develop low-cost, easily assembled, and locally manufactured ventilators. The ATENA ventilator project was developed in a community-based approach targeting the development, prototyping, testing, and decentralized manufacturing of a new mechanical ventilator.

**Objective:** This article aims to demonstrate ATENA's adequate performance and safety for clinical use.

**Material:** ATENA is a low-cost ventilator that can be rapidly manufactured, easily assembled, and locally produced anywhere in the world. It was developed following the guidelines and requirements provided by European and International Regulatory Authorities (MHRA, ISO 86201) and National Authorities (INFARMED). The device was thoroughly tested using laboratory lung simulators and animal models.

**Results:** The device meets all the regulatory requirements for pandemic ventilators. Additionally, the pre-clinical experiences demonstrated security and adequate ventilation and oxygenation, *in vivo*.

**Conclusion:** The ATENA ventilator had a good performance in required tests in laboratory scenarios and pre-clinical studies. In a pandemic context, ATENA is perfectly suited for safely treating patients in need of mechanical ventilation.

## Introduction

The global pandemic Coronavirus Disease 2019 (COVID-19) caused by SARS-CoV-2 created an urgent need for mechanical ventilators around the world (1). Even though the majority of patients develop mild (40%) or moderate (40%) symptoms, ~15% develop a severe condition that requires oxygen support and 5% end up with respiratory failure and need intensive care admission (2–4).

The exponential pattern of viral transmission led to a rapid and overwhelming increase in hospitalizations and overflow to intensive care units for invasive ventilation. At the beginning of 2020, the number of available ventilators was scarce and contributed to a significant increase in morbimortality worldwide (5–7). The fight against the global COVID-19 pandemic required innovative actions. Globally, thousands of experts, companies, and volunteers worked to fill the global shortage of commercial ventilators, by developing open-source ventilators or finding strategies (8–12) for shared ventilation (13, 14). Likewise, in Portugal, CEiiA—an Engineering and Product Development Centre—led (15) the development of the ATENA ventilator in a community-based approach.

ATENA is a rapidly manufactured, low-cost, easily assembled, and locally produced mechanical invasive ventilator. It was developed in a short time, from design to prototype. The requirements for the ATENA ventilator were common to other proposals (11, 16): (i) easily sourced components available to the general public; (ii) “open-source” compatibility, namely, availability of design and easiness to replicate; and (iii) high accuracy in a range of ventilation strategies that allow high airway pressures for ARDS patients.

This paper aims to demonstrate the performance and safety of ATENA as a pandemic ventilator adequate for COVID-19 patients, following the requirements of different regulatory agencies.

## Materials and Methods

### ATENA Design and Systems Breakdown

#### Overview

ATENA is a pneumatic ventilator, requiring high-pressure air and oxygen supplies to drive the respiratory cycles. Its control system hardware was locally designed, based on commercially available and inexpensive components. The software was developed in-house.

**Figure 1:**
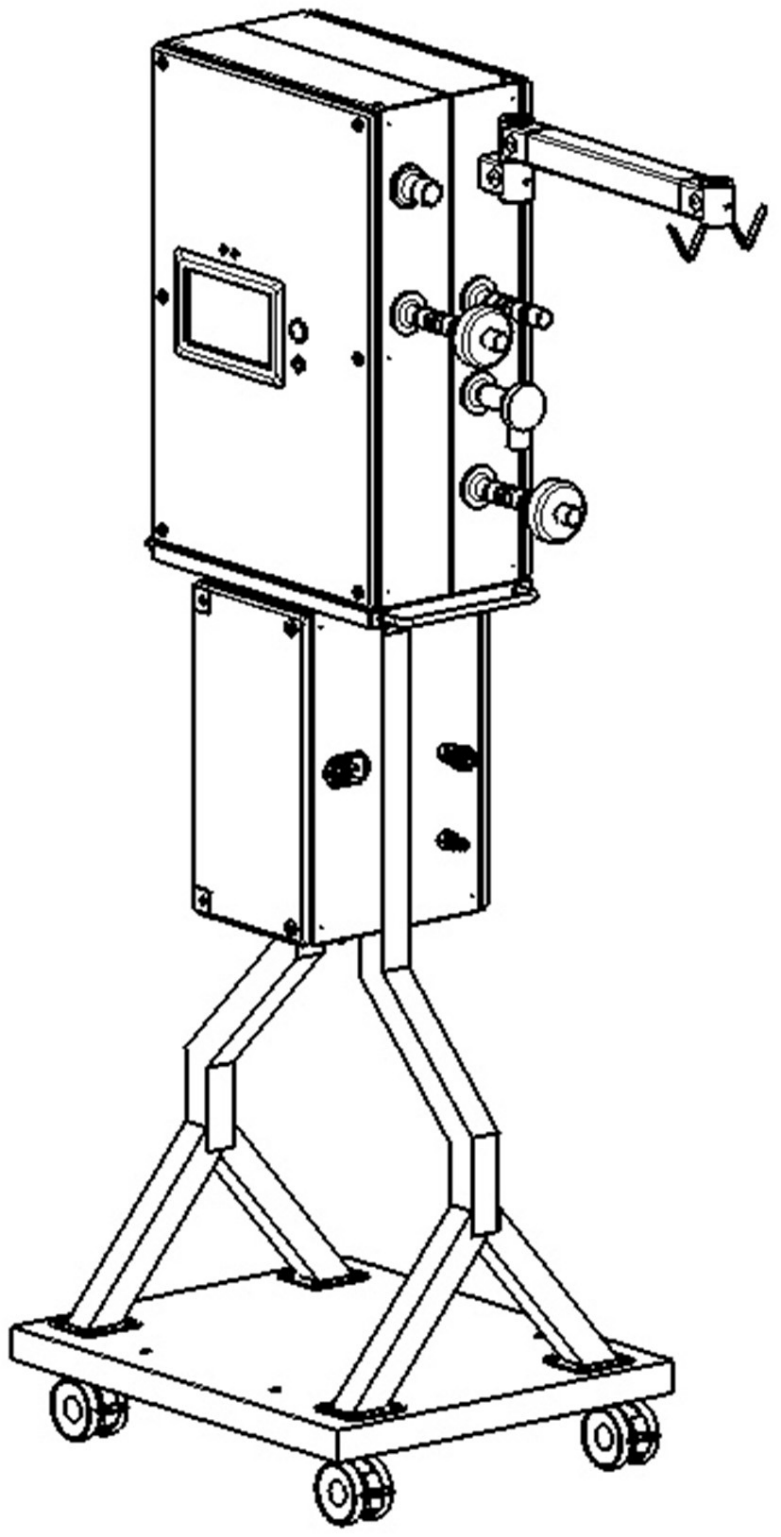
Fully mature manufactured ATENA Ventilator unit.

The main body is built using a stainless-steel wheeled structure and four sealed metal industrial boxes. These boxes are attached on each side of the structure (see Figure 1). They contain the pneumatic, electrical, and control modules. ATENA's overall specifications are described in Table 1 (see also Supplementary Table 1).

**Table 1:** ATENA's overall specifications and control ranges.

**Physical characteristics**	
Length	600 mm (23.6 in)
Width	500 mm (19.68 in)
Height	1,685 mm (66.3 in)
Weight	53.4 kg
**Electrical specifications**	
Input power	100–240 Vac (50–60 Hz) 30 W
Battery	Lead-acid battery Nominal voltage−12 V 7 Ah capacity
Usual charge/maintenance time	4 h/10 h
Battery autonomy	Up to 5 h
Sound Pressure Level	40 dB(A) ± 10 dB(A)
**Pneumatic specifications**	
Medicinal gases	Air and oxygen
Input pressure rated range	3 to 5 bar (300 to 500 kPa)
Maximum transient flow rate (3 s average)	120 L/min (Air: 60 L/min Air) (O2: 60 L/min O_2_)
Maximum input flow rate (10 s average)	Air: 35 L/min @ 280 kPa O_2_: 35 L/min @ 280 kPa
**Control specifications**	
Oxygen	21 to 100%
PEEP	0 to 40 cm H2O (0 to 39.2 hPa)
I:E	1:1–1: 4
Breathing rate (RR)	5 to 30 r/min
Peak Inspiratory Pressure (above PEEP)	0 to 40 cm H2O (0 to 39.2 hPa)
Tidal Volume	250–800 ml
Inspiration Pause Time (in% Ti)	0 to 50%
Assistance activation flow	0.2 to 3 L/min
FiO_2_ 21 to 90% response time	≤65 s
**Equipment classification**	
Protection class	Class I (Chassis earth connected)
Applied parts classification	Type B (Applied parts in contact with patient are not conductive)
Operation mode	Continuous
Mobility	Mobile Equipment (Can be wheeled but not during operation)
IP protection	IP22 The enclosure of the ventilator is protected against the ingress of solid objects bigger than 12, 5 mm and the ingress of liquids dripping when tilted at 15°.

The two upper boxes contain the respiratory circuit, with the required valves and sensors, and the control system. Gases in the respiratory circuit are at low pressure (<60 cmH_2_O). One of the lower housing boxes handles the input of air and oxygen at high pressure (>3 bar). The other contains the power module, composed of a battery, transformers, and safety fuses and breakers.

A touch screen displays pressure, flow, and volume curves in addition to other important ventilation variables (see Figures 3A,C). Ventilation modes and parameters are configurable *via* the touch screen (Figure 3B). Operation is started/stopped by pressing a physical button on the front of the ventilator.

Alarms are signaled by sound, light, and a specific alarm text on the display (see the top bar in Figure 3A). Alarms need to be acknowledged and muted by pressing another physical button.

#### Operation

Ventilation mode and parameters are configured *via* the touch display. Figure 3B shows the VCV (volume-controlled ventilation) mode configuration screen. Other modes are visible as extra tabs. Once the operation mode is selected and configured, ventilation can be started by pressing the START/STOP button next to the display.

**Figure 2:**
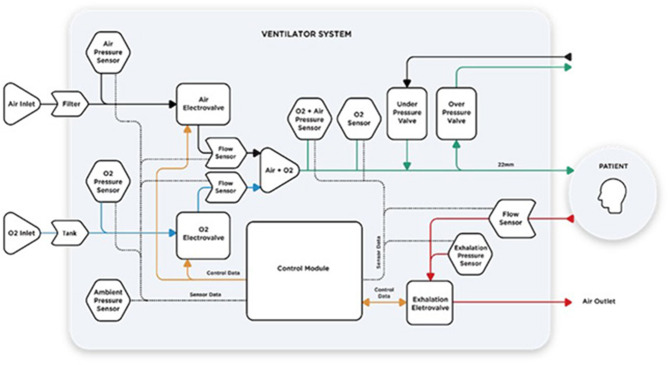
ATENA High-Level Systems Architecture.

**Figure 3:**
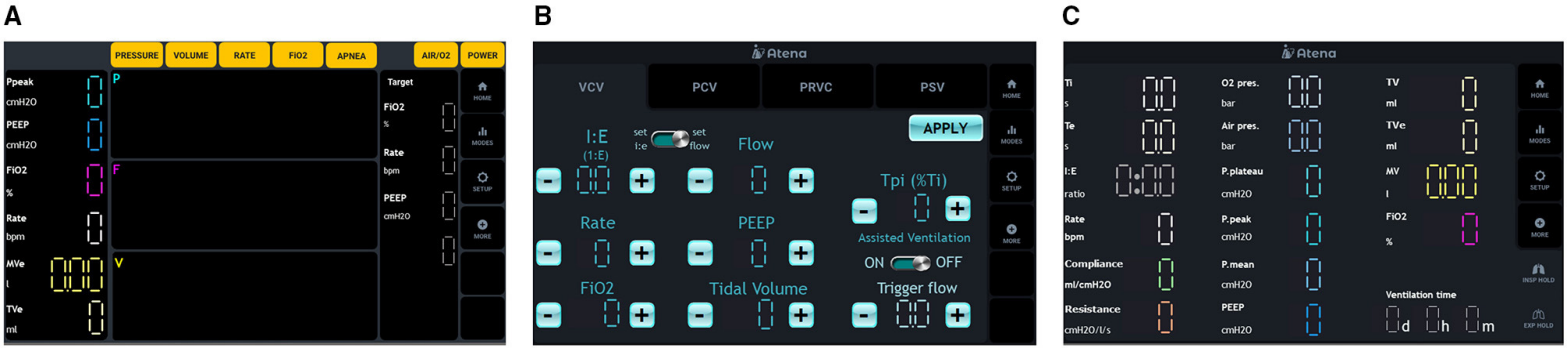
ATENA Graphical User Interface. **(A)** Left panel, **(B)** central panel, and **(C)** right panel.

Air and oxygen enter the system at a pressure of about 4.5 bar (air and O_2_ inlets are indicated in Figure 2). To ensure adequate availability of oxygen for each inhalation, we installed a 0.75-L buffer tank after the oxygen inlet. Pressure is monitored *via* pressure sensors in each of the high-pressure circuits to detect any fault in the gas supply. Such faults trigger an alarm. These pressures can be checked on the ventilator display (Figure 3C).

Piezo-electric valves on the air and oxygen lines control the flow and oxygen content of the mixture delivered to the patient. The control system uses two flow sensors, one for each gas, for feedback control loops. An oxygen cell measuring the fraction of inspired oxygen (FiO_2_) allows for mixture correction. Data from pressure sensors on both inspiratory and expiratory circuits are used to estimate the airway opening pressure. Depending on the mode of ventilation, the control system will deliver a constant flow, or variable flow, to reach a set pressure. All the aforementioned sensed variables are shown on the ventilator display (Figures 3A,C).

The gas mixture leaves the ventilator through a 22-mm tube outlet, compatible with standard medical tracheas, check valves, and filters. Additionally, two safety mechanisms prevent exposure to overpressure (manually adjustable pressure relief valve, maximum 60 cmH_2_O) and suffocation (one-way under-pressure valve). The connection ports for the overpressure and anti-suffocation valves are also standard 22-mm tubes. Under normal operating conditions, the control system will prevent those conditions; the safety mechanisms are redundant systems.

To allow for both an unobstructed exhalation and fine control of flow during the PEEP phase, we selected a pneumatically controlled pinch valve. This valve has a large internal diameter, capable of large flows even in low-pressure conditions. During inhalation, the exhalation valve is closed. At the start of the exhalation, the valve fully opens until the pressure reaches the configured PEEP value. While keeping the PEEP, the exhalation valve is slightly open so that a small bias flow is present. This bias flow is useful for detecting an attempt by the patient to initiate a breath. A flow sensor in the exhalation circuit, in combination with both flow sensors in the inhalation circuit, lets us estimate the actual flow from/to the patient.

Spontaneous breaths are detected by both a variation in pressure and a flow toward the patient during the exhalation period. We found this combined approach to be more robust than a single breath variable measurement.

### Requirements and Specifications

ATENA was developed following the clinical requirements for the “minimally acceptable” performance of a mechanical ventilator aimed at the COVID-19 pandemic crisis. Those requirements and specifications were described in the Rapid Manufactured Ventilator System (RMVS) document by the UK Medicines & Healthcare products Regulatory Agency (MHRA) (17) and in the exceptional authorization by the National Authority for Drugs and Healthcare Products (deliberation of 29 June 2020, INFARMED) (18).

The ATENA ventilator can operate in continuous mandatory ventilation (pressure-regulated volume control and pressure- and volume-controlled modes) and in pressure support mode. ATENA's overall specifications and control ranges are presented in Table 1.

### Testing Protocol

The main objective of the testing protocol was to demonstrate adequate safety and performance by the ATENA ventilator. We evaluated ATENA's accuracy and performance in five steps: (1) MHRA protocol, including endurance testing (17); (2) International Organization for Standardization (ISO 80601-2-12:2020) (19); (3) additional tests for extreme values of tidal volume, PEEP, and FiO_2_; (4) pressure support ventilation; and (5) pre-clinical studies.

ATENA was attached *via* the breathing circuit to a calibrated electronic gas flow analyzer ventilator tester (VT900A, Fluke Biomedical) and then to an adult test lung (ACCU LUNG™ Precision test, Fluke Biomedical) that simulates different respiratory systems with variable compliance (C) and resistance (R). The ventilator was also linked to an external computer that allowed the recording of pressure, flow, FiO_2_, and volume waveforms.

#### MHRA Protocol

The assessment of the performance of ATENA was done under controlled conditions following MHRA's RMVS protocol (17). We performed three sets of 36 trials for VC: (i) compliance, (ii) resistance, and (iii) tidal volume, and two sets of 36 trials for PC: (i) plateau pressure at 15 cmH_2_O and (ii) 30 cmH_2_O.

Concerning endurance testing, ATENA was connected to a passive lung simulator for 24 h. Ventilator settings at the beginning and the end of the test were reported, and the variation of the settings was analyzed.

Electromagnetic compatibility, electrical interference, emission, and immunity tests were made following European and international standards (EN 60601-1-2, EN 61000-6-1, EN 61000-4-4, EN 61000-4-6, EN 55011, and ISO 80601-2-12).

#### International Organization for Standardization

Simultaneously, the ATENA ventilator was submitted to ISO requirements testing for basic safety and essential performance of critical care ventilators (ISO 80601-2-12:2020) (19). For volume-controlled ventilation, seven tests were done with variable compliance and resistance lung models, using different tidal volume, PEEP, and FiO_2_. To evaluate the performance of pressure-controlled ventilation, seven tests were done also with different compliance and resistance levels, using different airway pressure, PEEP, and FiO_2_. Additionally, one set of 14 tests was done to assess ATENA's accuracy in the measurement of tidal volume.

#### Additional Tests

Following recommendations from INFARMED (19), ATENA performed an additional set of tests with a simulated ARDS lung model (C = 20 ml/cmH_2_O and R = 5 cmH_2_O/L/s). This allowed us to evaluate the accuracy of the ventilator with an extreme variation of tidal volume (250–800 ml) in VCV and of PEEP (0–40 cmH_2_O) in PCV modes. We ran an extra test with a healthy lung model, to analyze ATENA's accuracy on FiO_2_ control (21–100%). Those tests were done without pausing the ventilator, to evaluate ATENA's response to a change in configuration parameters. The same breathing simulator and gas flow analyzer were used.

#### Pressure Support Ventilation

To evaluate the performance of the pressure support ventilation mode, we used an ASL5000 breathing simulator (version 3.6, Active Servo Lung, IngMar Medical, Pittsburgh, PA, USA) with an active lung model in both healthy and ARDS conditions. The settings used for each healthy (C = 50 ml/cmH_2_O and R = 10 cmH_2_O/L/s) and ARDS patient (C = 30 ml/cmH_2_O and R = 20 cmH_2_O/L/s) were available from the simulator's patient library and were validated in previous studies (11, 20–22).

ASL5000 tested ATENA's (i) capacity to detect apnea and switch to backup controlled mandatory ventilation (pressure-controlled ventilation) and (ii) performance on the pressure support mode with increasing inspiratory muscle strength. Given that the ATENA ventilator has an inspiratory trigger flow with a sensitivity that varies between 0.2 SLPM (high sensitivity) and 3 SLPM (low sensitivity), we decided to test three trigger points (0.2, 1, and 2 SLPM). For both healthy and ARDS lung models, the same spontaneous breathing parameters were used (respiratory rate: 3/15 cpm; uncompensated residual capacity: 0.5 L; pause: 0%; inspiratory muscle pressure: 0/10/15 cmH_2_O; expiratory muscle pressure: 0 cmH_2_O; inspiratory rise time: 10%; inspiratory hold: 5%; inspiratory release time: 10%; expiratory rise time: 0%; expiratory hold: 0%; expiratory release time: 0%; effort: sinusoidal). A total of 21 tests were performed on pressure support of 10 cmH_2_O with a PEEP of 5 cmH_2_O for testing ATENA's capacity to support the weaning process from mechanical ventilation (23, 24).

#### Pre-Clinical Test

Pre-clinical tests were performed following the EU Directive 2010/63/EU, approved by the Animal Welfare Body (ORBEA EM/ICVS-I3Bs_005/2020) of the institution where the study was conducted (University of Minho) and by the national authority for animal protection—*Direção Geral de Alimentação e Veterinária* (DGAV 008337). Four porcine animals (*Sus scrofa domesticus*) (14 weeks; average weight 32 ± 3 kg) were used to evaluate ATENA's performance *in vivo*, with a special focus on blood gas exchange during the MRHA test protocol. Two animals were allocated to the volume-controlled (group 1), and two animals were allocated to the pressure-controlled (group 2) ventilation. Animals were anesthetized with an intramuscular administration of ketamine (20 mg/kg) and xylazine (2 mg/kg), followed by intravenous propofol administration (4 mg/kg). Total Intravenous Anesthesia (TIVA) was maintained with continuous propofol infusion (4.4 mg/kg) in combination with fentanyl (0.005 mg/kg/h) and midazolam (0.7 mg/kg/h) administered through a central venous catheter, together with the parenteral isotonic fluids administered for maintenance of water and electrolyte balance (6–10 ml/kg/h). Rocuronium (2.5 mg/kg/h) was administered to provide muscle relaxation and improve ventilator adaptation. After intubation, animals were adapted to the ATENA ventilator with a FiO_2_ of 60%. An invasive arterial line was achieved for a continuous hemodynamic (Combitrans Monitoring-Set arterial, BBraun, Germany) and blood gas (CG4+ cartridges, i-Stat analyzer, Abbott, Chicago, IL) monitoring.

In both groups, after 5 min of ventilation, arterial blood gas was collected to measure and record pH, PO_2_ and PCO_2_, and HCO_3_, and a new ventilatory setting was selected. At the end of the experiment, with the animals still under deep anesthesia, euthanasia was performed by an intravenous administration of pentobarbital (200 mg/kg), with veterinary support.

## Results

### MHRA Protocol

In volume-controlled ventilation, a triplet of 36 trials for compliance, resistance, and tidal volume were done. In pressure-controlled ventilation, the same number of tests were completed for PEEP, plateau pressure, and FiO_2_ for an inspiratory pressure of 15 cmH_2_O or 30 cmH_2_O.

**Figure 4:**
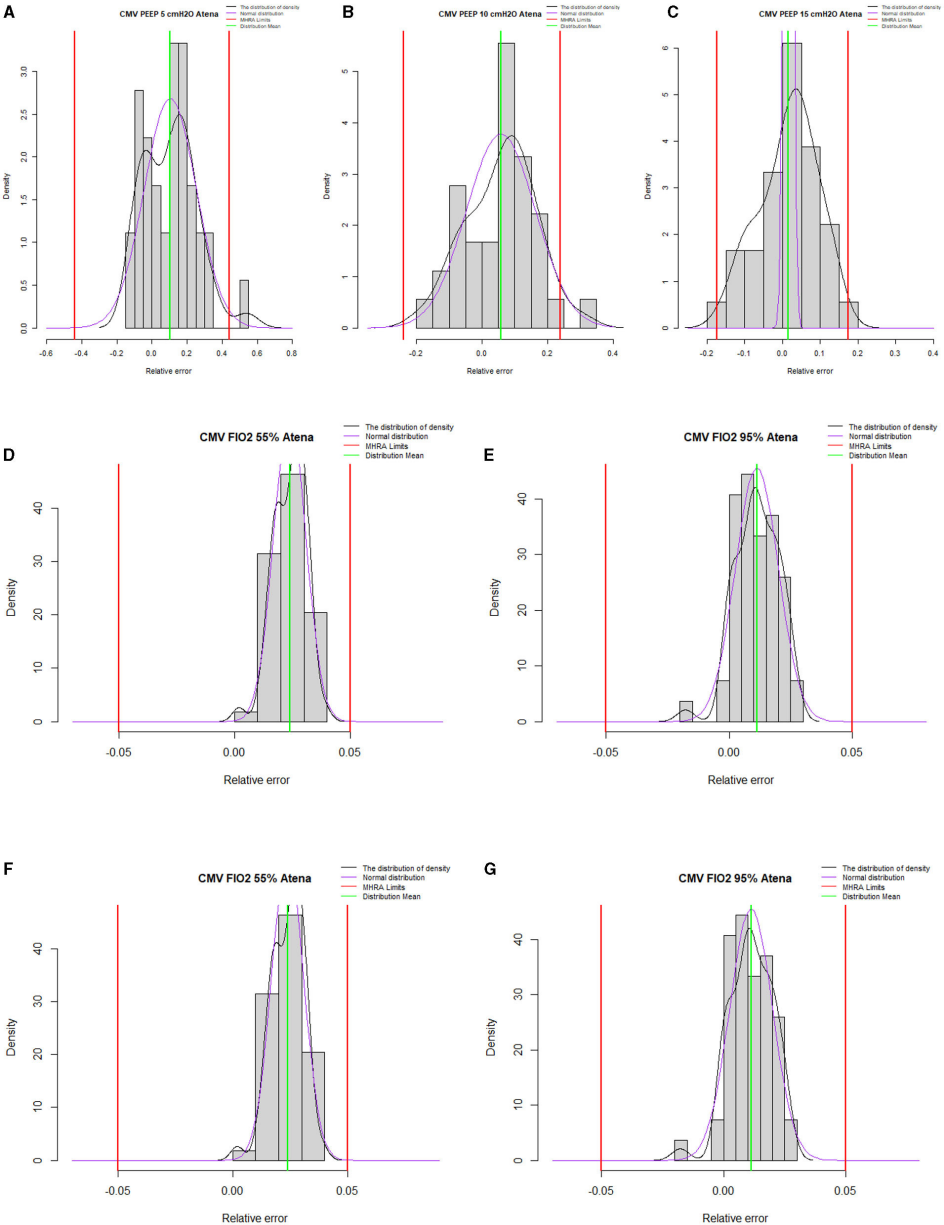
Summary histograms for all volume-controlled ventilation test conditions. **(A–C)** represent histograms for PEEP 5, 10, and 15, respectively. **(D,E)** represent histograms for tidal volume 500 and 300 ml, respectively. **(F,G)** represent histograms for 55 and 95% of FiO_2_. Outside the red limits data points out-of-performance.

**Figure 5:**
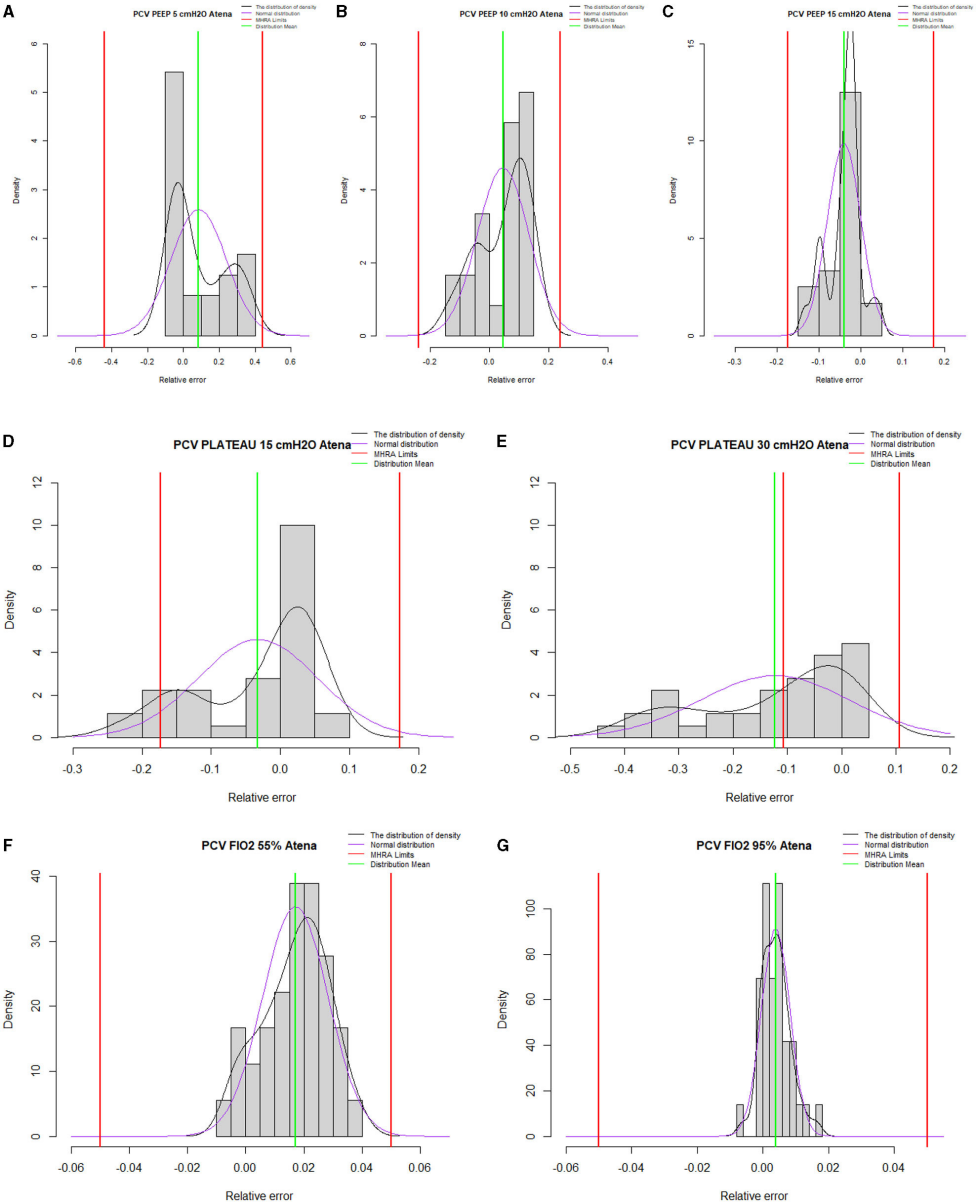
Summary histograms for all pressure-controlled ventilation test conditions. **(A–C)** represent histograms for PEEP 5, 10, and 15 cmH_2_O, respectively. **(D,E)** represent histograms for plateau pressure of 15 and 30 cmH_2_O, respectively. **(F,G)** represent histograms for 55 and 95% of FiO_2_. Outside the red limits data points out-of-performance.

The histograms in Figures 4, 5 represent the relative frequency of the error (%) in volume-controlled ventilation for different PEEP values (5, 10, and 15 cmH_2_O); tidal volume (300–500 ml) and FiO_2_ (55–95%) and pressure-controlled ventilation for different PEEP values (5, 10, and 15 cmH_2_O); and plateau pressure (15–30 cmH_2_O) and FiO_2_ (55–95%).

Concerning endurance testing, ATENA ran uninterrupted for 24 h, which represents 28,800 cycles. In volume-controlled ventilation, the set at the beginning of the test was as follows: tidal volume of 500 ml, respiratory rate 20 breaths/min, PEEP 5 cmH_2_O, and FiO_2_ 40%. The average of measured parameters in the first 13 min was a tidal volume of 512.73 ml, FiO_2_ = 39.81%, and PEEP = 4.66 cmH_2_O. In the last 13 min of the 24 h, the average of the values measured were as follows: tidal volume = 519.57 ml, O2 = 39.72%, and PEEP = 5.47 cmH_2_O at the final (time = 24 h).

For the pressure-controlled ventilation mode, a goal was set for the plateau pressure of 20 cmH_2_O, respiratory rate of 20 breaths/min, PEEP 5 cmH_2_O, and FiO_2_ 40%. The average values measured after 13 min of ventilation were as follows: plateau pressure = 22.61 cmH_2_O, O_2_ = 39.81, and PEEP = 4.47 cmH_2_O. In the last 13 min of 24 h of ventilation, the average values were as follows: plateau pressure = 22.61 cmH_2_O, O_2_ = 40.3%, and PEEP = 4.81 cmH_2_O.

The relative error values measured between the initial and the last 13 min are tidal volume = 1.3%, FiO_2_ = 0.2%, and PEEP = 7.4% for controlled ventilation and plateau pressure = 0%, O_2_ = −1.2%, and PEEP = −7.6%.

In terms of electronic safety, the ATENA ventilator complies with the relevant requirements studied (EN 60601-1-2, EN 61000-6-1, EN 61000-4-4, EN 61000-4-6, EN 55011, and ISO 80601-2-12).

### International Organization of Standards

ATENA fulfilled the following criteria for basic safety and essential performance of critical care ventilators (ISO 80601-2-12:2020):

- Maximum error of the delivered and monitored tidal volume compared to the set value: ± (4 ml + 15% of the set value);
- Maximum error of the PEEP compared to the set value: ± (2 cmH_2_O + 4% of the set value);
- Maximum error of FiO_2_ compared to the set value: ± (5% of the set value); and
- Maximum error of P_Plateau_ compared to the set value: ± (2 cmH_2_O + 4% of the set value).

Relevant data from volume- and pressure-controlled ventilation, as well as expired volume, measured criteria are presented in Supplementary Tables 2, 3.

### Additional Tests

**Figure 6:**
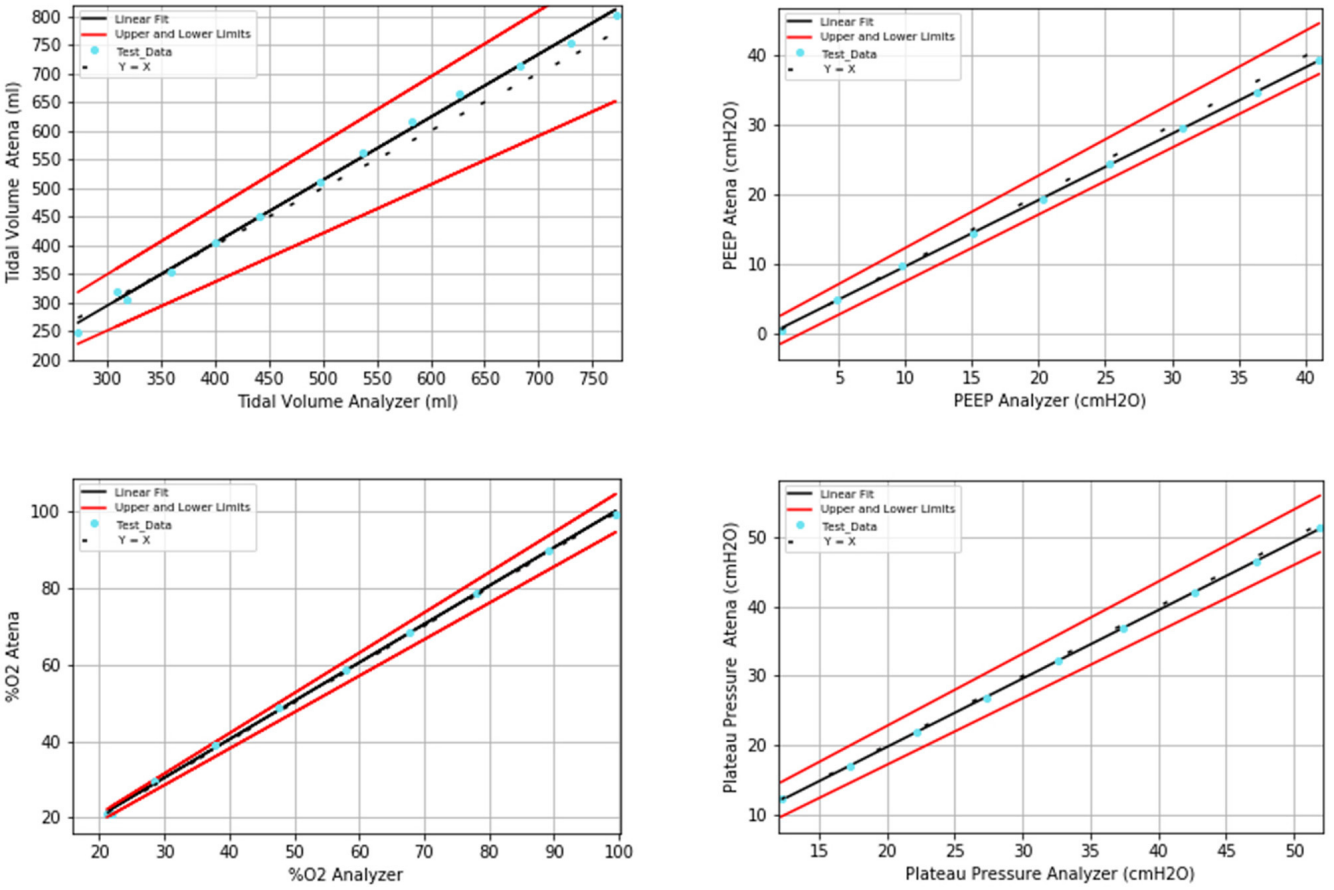
Linear-fit results from additional tests for a wide range of settings (tidal volume, PEEP, FiO_2_, and plateau pressure, respectively).

The results of additional tests are presented in Figure 6. The data are presented and analyzed in terms of relative error between the values defined by the test conditions, configured in ATENA, and the values measured by the gas flow analyzer. The relationship is linear for all evaluated parameters (tidal volume, PEEP, FiO_2_, and P_Plateau_).

### Pressure Support Ventilation

The results obtained in tests 1 to 21 show that ATENA had an adequate performance for all trigger levels, for all inspiratory muscle pressures used in both healthy and ARDS simulated lungs (see Supplementary Table 4).

### Pre-Clinical Test

During the study period, all animals remained hemodynamically stable with no need for vasopressor support. Supplementary Tables 5, 6 display the relevant ventilatory settings and measures of ventilation variables in pre-clinical testing for volume- and pressure-controlled modes, respectively.

## Discussion

### Analysis of Results

Test results demonstrated that the ATENA ventilator met the specifications in the MHRA RMVS guidelines. Its accuracy in terms of maximum bias and linearity errors is inside the performance limits for ISO 80601-2-12-2020 requirements.

ATENA can achieve satisfactory performances for all volume-controlled ventilation and pressure-controlled ventilation test conditions with a plateau pressure of 15 cmH_2_O. Limited performance was observed in only three tests for plateau pressure of 30 cmH_2_O, which explains the outlier values presented in Figure 5:

#1 and 2–In the test with a plateau pressure of 30 cmH_2_O, compliance of 50 ml/cmH_2_O, resistance of 5 cmH_2_O/L/s, and respiratory rate of 20 cpm, both plateau pressure and PEEP were almost achieved. A high instantaneous flow (SLPM) would be required for the PEEP and plateau pressure setpoints to be reached. In fact, during these tests, tidal volumes as high as 1,000 ml were recorded. For a desired FiO_2_ of 95%, the required instantaneous flow would exceed the maximum capabilities of ATENA's hardware. Due to the high FiO_2_ setpoint, a single piezo-electric valve, the oxygen one, would limit the maximum flow to its capacity, ~60 SLPM. It is important to notice that ATENA contains two piezo-electric valves, one for air and one for oxygen, each rated to a maximum of 60 SLPM. Again, the tidal volume observed in these tests is not adequate for the protective ventilation that is needed for COVID-19 patients.
#3–For a plateau pressure of 30 cmH_2_O, compliance of 50 ml/cmH_2_O, and FiO_2_ set to 95%, this last parameter could not be reached. This limitation is once again explained by ATENA's hardware capabilities that, when mostly using a single piezo-electric valve, are capable of reaching instantaneous flows of 60 SLPM.

The majority of ISO requirements were met by ATENA. However, for the accuracy tests of volume-controlled ventilation (Supplementary Table 2, tests 1 and 2), in a simulated healthy patient (C = 50 ml/cmH_2_O and linear R of up to 20 cmH_2_O/L/s, tidal volume 500 ml), the PEEP parameter was underperforming. These results were considered to be sufficient for the adequate validation of ATENA since it is not expected that patients who developed bilateral pneumonia due to COVID-19 with the need for invasive ventilation (the scope of use for the ATENA ventilator) present a lung compliance of 50 ml/cmH_2_O (25). Furthermore, ATENA displays the accurately measured PEEP, so even if underperforming, it will provide the clinician the required data to make an informed decision.

Simultaneously, the analysis of the small PEEP coefficients of variation in tests performed in volume- and pressure-controlled ventilation allows us to affirm the stability and safety of this parameter in situations that mimic clinical practice.

Taking into account all of the above, it is possible to conclude that the ATENA adequately performed for all test conditions set by the MHRA's RMVS and ISO guidelines, except for a single set of conditions not commonly found in day-to-day clinical practice and may therefore be ruled as less relevant for a pandemic COVID-19 ventilator.

Results from the additional tests that extended the setting parameters of tidal volume, PEEP, FiO_2_, and plateau pressure to their extreme limits prove that ATENA can perform adequately in an enlarged range of values.

We studied ATENA's performance on pressure support (PS) ventilation mode with the settings commonly used for weaning a patient from the ventilator (pressure support of 10 cmH_2_O and PEEP of 5 cmH_2_O). The analysis of the results in these settings revealed that ATENA was able to provide PS ventilation in the range of flow trigger values under study and its performance was not impaired when we assessed different levels of inspiratory muscle strength, in both healthy and ARDS lung models.

Noteworthy, even though two HME/HEPA filters were included on the 1.5-m breathing tubes, during the pressure support tests, the muscle pressure required to successfully trigger the ventilator was adequate. This indicates that the trigger sensitivity is robust and is adapted to real working conditions.

During *in vivo* animal model studies, ATENA behaved within expectations for the required standards across the full range of configurations, in both volume- and pressure-controlled modes, maintaining excellent oxygenation and ventilation performance. In both groups, changes in partial gas pressure and arterial pH secondary to changes in ventilatory parameters were biologically expected. In no trial was oxygenation compromised, and an improvement in the partial pressure of oxygen in arterial blood in response to the increase in ventilation/min was always observed. At the same time, the hyperventilation that conditioned the presence of respiratory alkalosis in numerous blood gases assertively demonstrates the effectiveness of ATENA in ventilation.

## Conclusions

The novel ATENA Medical Ventilator, here described, was extensively tested, fulfilling MHRA and ISO requirements and specifications. Additional tests for extended parameter limits, pressure support, and pre-clinical studies demonstrated that it can perform as required. The ventilator is capable of controlled modes VCV, PCV, and PRVC, and mode PSV, and is therefore entirely appropriate for clinical use in adult COVID-19 patients. Further clinical trials must be performed to ultimately and unequivocally validate ATENA in clinical practice.

## Data Availability Statement

The raw data supporting the conclusions of this article will be made available by the authors, without undue reservation.

## Ethics Statement

The animal study was reviewed and approved by EU Directive 2010/63/EU, under project authorization attributed by local ethics committee (Subcomissão de Ética para as Ciências da Vida e da Saúde - SECVS 004/2016, Chairperson Prof. Cecília Leão) and by the National Authority for animal protection (Direção Geral de Alimentação e Veterinária - et al. DGAV 015296, Chairperson Dr. Fernando Bernardo).

## Author Contributions

TR, FB, HO, JM, JA, JL, JS, MO, PF, RaM, and RuM: CEiiA's engineering team and were responsible for conceiving, developing, prototyping, and testing the ventilator throughout its development and validation. EN, EA, HM, JP, NS, PB, and SG: medical core team and Defined the specifications, tested, and validated the ATENA ventilator throughout its development and validation phases. TR, JA, SG, and JP: study conception and design of the paper. TR, JA, FB, PF, JP, RaM, and SG: acquisition of data, analysis and interpretation of data, and drafting the article. TR, JA, SG, RaM, and JP: in-depth revising the manuscript critically for important intellectual content. All authors contributed to the study and final approval of the version to be published and all agree to be accountable for all aspects of the work, thereby ensuring that questions related to the accuracy or integrity of any part of the work are appropriately investigated and resolved.

## Conflict of Interest

The authors declare that the research was conducted in the absence of any commercial or financial relationships that could be construed as a potential conflict of interest.

## Publisher's Note

All claims expressed in this article are solely those of the authors and do not necessarily represent those of their affiliated organizations, or those of the publisher, the editors and the reviewers. Any product that may be evaluated in this article, or claim that may be made by its manufacturer, is not guaranteed or endorsed by the publisher.

## Supplementary Material

Supplementary Table 1 Description of each ATENA's component and the corresponding manufacturer.

Supplementary Table 2 ATENA accuracy of volume and pressure-controlled ventilation (ISO 80601-2-12, clause 201.12.1.102).

Supplementary Table 3 ATENA accuracy of expired volume indication (ISO 80601-2-12, clause 201.12.4.103.1).

Supplementary Table 4 Results from ASL5000, on pressure support ventilation in healthy and ARDS lung model.

Supplementary Table 5 Relevant ventilatory settings and measures of ventilation (average ± standard deviation) in pre-clinical testing for volume-controlled ventilation. TV—tidal volume; RR—respiratory rate; PEEP—positive end-expiratory pressure; PO_2_–oxygen arterial partial pressure; PCO_2_–CO_2_ arterial partial pressure; ${\text{HCO}}_{3}^{-}$–bicarbonate arterial concentration.

Supplementary Table 6 Relevant ventilatory settings and measures of ventilation (average ± standard deviation) in pre-clinical testing for pressure-controlled ventilation. TV—tidal volume; RR—respiratory rate; PEEP—positive end-expiratory pressure; PO_2_–oxygen arterial partial pressure; PCO_2_–CO_2_ arterial partial pressure; ${\text{HCO}}_{3}^{-}$–bicarbonate arterial concentration.
