# Supplementary figures and images for: ATENA–A Novel Rapidly Manufactured Medical Invasive Ventilator Designed as a Response to the COVID-19 Pandemic: Testing Protocol, Safety, and Performance Validation
Source: Front Med (Lausanne). 2021 Aug 19;8:614580. doi: 10.3389/fmed.2021.614580 (PMC8418230; doi:10.3389/fmed.2021.614580)

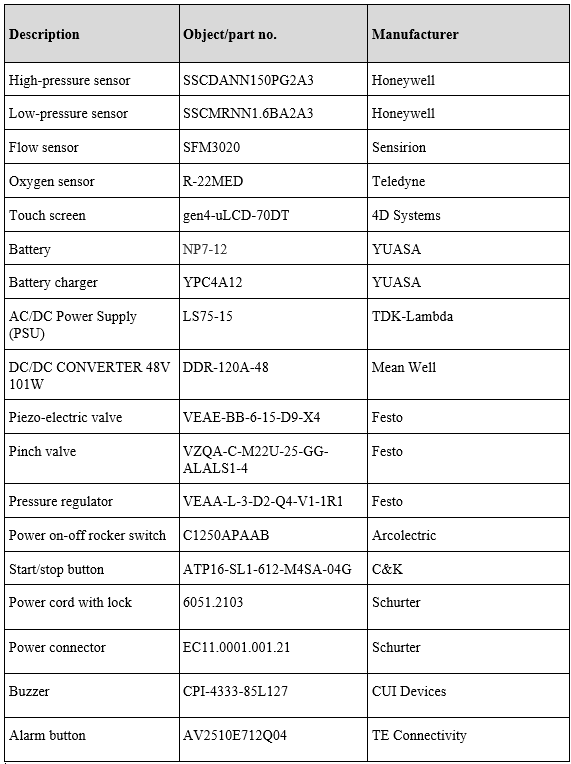

Supplement: Supplementary Table 1 — Description of each ATENA's component and the corresponding manufacturer. [file Image_1.PNG]

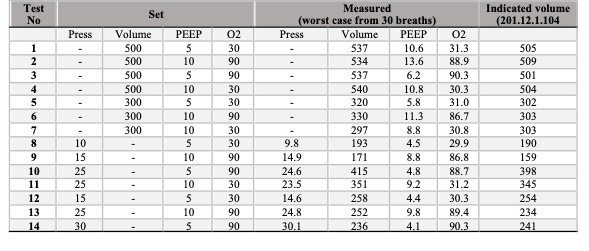

Supplement: Supplementary Table 2 — ATENA accuracy of volume and pressure-controlled ventilation (ISO 80601-2-12, clause 201.12.1.102). [file Image_2.JPEG]

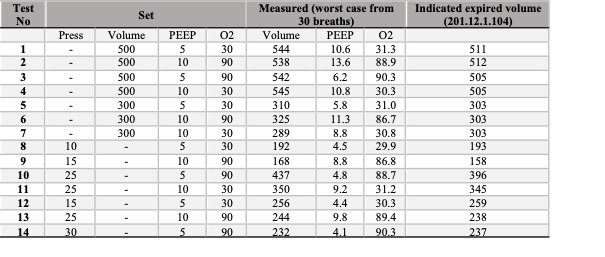

Supplement: Supplementary Table 3 — ATENA accuracy of expired volume indication (ISO 80601-2-12, clause 201.12.4.103.1). [file Image_3.JPEG]

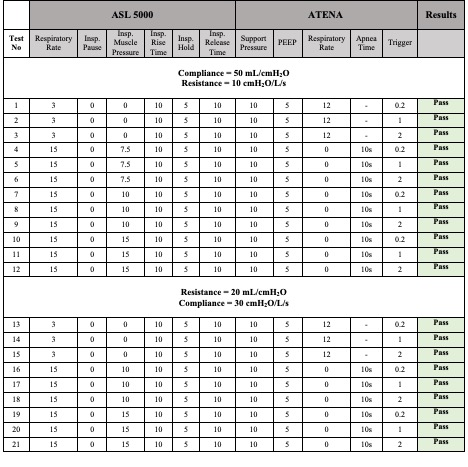

Supplement: Supplementary Table 4 — Results from ASL5000, on pressure support ventilation in healthy and ARDS lung model. [file Image_4.JPEG]

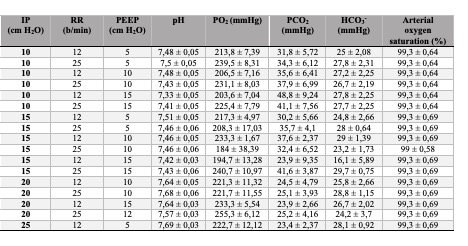

Supplement: Supplementary Table 5 — Relevant ventilatory settings and measures of ventilation (average ± standard deviation) in pre-clinical testing for volume-controlled ventilation. TV—tidal volume; RR—respiratory rate; PEEP—positive end-expiratory pressure; PO2–oxygen arterial partial pressure; PCO2–CO2 arterial partial pressure; HCO3-–bicarbonate arterial concentration. [file Image_5.JPEG]

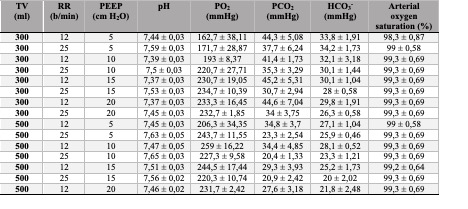

Supplement: Supplementary Table 6 — Relevant ventilatory settings and measures of ventilation (average ± standard deviation) in pre-clinical testing for pressure-controlled ventilation. TV—tidal volume; RR—respiratory rate; PEEP—positive end-expiratory pressure; PO2–oxygen arterial partial pressure; PCO2–CO2 arterial partial pressure; HCO3-–bicarbonate arterial concentration. [file Image_6.JPEG]
